# Preparing Copper Nanoparticles and Flexible Copper Conductive Sheets

**DOI:** 10.3390/nano12030360

**Published:** 2022-01-23

**Authors:** Gui-Bing Hong, Jia-Fang Wang, Kai-Jen Chuang, Hsiu-Yueh Cheng, Kai-Chau Chang, Chih-Ming Ma

**Affiliations:** 1 Department of Chemical Engineering and Biotechnology, National Taipei University of Technology, Taipei 10608, Taiwan; lukehong@ntut.edu.tw (G.-B.H.); d8906002@mail.ntust.edu.tw (J.-F.W.); 2 School of Public Health, College of Public Health and Nutrition, Taipei Medical University, Taipei 11490, Taiwan; 3 Department of Public Health, School of Medicine, College of Medicine, Taipei Medical University, Taipei 11490, Taiwan; 4 Department of Nursing, St. Mary’s Junior College of Medicine, Nursing and Management, Yi-Lan 26647, Taiwan; noidea@smc.edu.tw (H.-Y.C.); amy03124@smc.edu.tw (K.-C.C.); 5 Department of Cosmetic Application and Management, St. Mary’s Junior College of Medicine, Nursing and Management, Yi-Lan 26647, Taiwan

**Keywords:** copper nanoparticles, chemical reduction, copper ink, flexible conductive sheets

## Abstract

Nanotechnology is used in a wide range of fields, including medicine, cosmetics, and new material development, and is one of the most popular technologies in the field of flexible electronic products. For the present work, the chemical reduction method with environmentally friendly reducing agents was used to synthesize copper nanoparticles (CuNPs) with good dispersibility. The CuNPs were characterized by transmission electron microscopy (TEM), X-ray diffraction (XRD), and ultraviolet–visible spectrophotometry (UV–vis). After the CuNPs were formed, the solvent, polymers, and additives were added to form copper ink. Finally, the prepared copper inks were applied to flexible polyethylene terephthalate (PET) substrate under low sintering temperature and the effects of sintering time and different concentrations of sintering agent on resistivity were investigated. The results show that the copper nanoparticles synthesized by secondary reduction were smaller, more uniform, and better dispersed than those formed by primary reduction. Ethylene glycol has reducing effects under high temperatures; therefore, the CuNPs formed using the mixed solvent were small and well dispersed. The copper ink was applied on the PET substrate, treated with a formic acid aqueous solution, and sintered at 130 °C for 60 min, and its resistivity was about 1.67 × 10^−3^ Ω cm. The proposed synthesizing method is expected to have potential applications in the flexible electronic products field.

## 1. Introduction

Flexible electronics have become increasingly small, thin, and lightweight, and have been incorporated into circuit boards, monitors, wearable devices, and electronic appliances [1,2]. Liquid metallic ink is directly printed onto flexible substrate, increasing the efficiency of manufacturing; in turn, the demand for nanometallic ink has increased [3,4]. Metallic nanoparticles have a larger ratio of surface area to volume than block materials do; therefore, compared with conventional materials, nanoparticles have many unique physical and chemical properties, as well as lower melting points, and can therefore be processed at relatively low temperatures [5]. Nanosynthesis methods can be divided into physical and chemical reduction methods [6,7,8]. Physical reduction methods are top-down, involving the use of grinding or laser cutting to miniaturize large metallic particles. Chemical reduction methods are bottom-up, involving oxidation reduction reactions that handle the precursor in the form of an atomic ion with a reducing agent in a suitable solvent [9,10]. The created metallic nanoparticles have various particle size distributions and stability levels based on conditions such as the reducing agent concentration, reduction potential, temperature, pH value, and stabilizer [11,12,13]. Chemical reduction methods are most commonly used for synthesizing various types of nanoparticles because they do not require special equipment and are relatively easy to conduct [14,15,16,17].

Typically, metallic nanoparticles are formed over three stages, the first of which is prenucleation. At this stage, because of changes in temperature or concentration, the metal cations in the metal salt are reduced to metal atoms that are randomly arranged and move freely while in the solution. The second stage is nucleation, in which the metal atoms in the solution continue to collide with one another, clustering to form nuclei. The final stage is growth, in which more solids are gradually deposited on the surface of the nucleus until the nucleus grows to a critical size, thereby forming a nanoparticle [5,18,19,20]. However, most of the chemicals employed in metallic nanoparticle preparation are toxic, and improper treatment of these chemicals could lead to environmental pollution. Therefore, the preparation of environmentally friendly, low-temperature-sintering metallic nanoparticles has become a research trend [21,22,23,24]. In past research [18,25,26], reports clearly demonstrated that ascorbic acid is a popular reducing agent. It is a natural and mild reducing agent with antioxidant properties and is widely used as a food preservative. It presents no biological hazards and its impact on the environment is minimal. However, ascorbic acid, with rather weak reducibility, requires a longer reduction time.

Silver, copper, and gold are the three most frequently studied metals for nanoparticles [10,11,24,27]. Silver and gold are costly. Copper, cheaper than silver and with equivalent conductivity, has received much attention. When nanonizing copper, its surface activation energy increases, causing it to display unique properties, including a low melting point, robust magnetism and light absorption, and material firmness [28,29,30]. Nanonized copper is used in conductive ink, catalysts, electronic products, antibacterial agents, nanoinjection molding, and humectants [14,31,32,33,34,35,36]. Therefore, the preparation of stable and antioxidant CuNPs is often the focus of research and development [37]. Unfortunately, copper (during synthesis) and CuNPs are easily oxidized, and when used in conductive ink, eliminating the oxidized layer on the surface of the CuNPs requires high sintering temperatures. Furthermore, studies on the preparation of CuNPs have often employed strong reducing agents, such as sodium borohydride or hydrazine, to create smaller particles or to reduce the reduction time [33]. However, these types of reducing agents are typically highly toxic, resulting in environmental pollution. However, weaker reducing agents have larger metal particles and require longer reduction times [38,39].

Applying CuNPs in conductive ink production requires a sintering step. The purpose of sintering is to melt the CuNPs into dense blocks through heat or pressure to achieve conductivity [25,35,40]. However, although this is known as low-temperature sintering, it typically involves temperatures as high as 160 °C. Conventional sintering processes typically involve high temperatures, which are energy-intensive and produce thermal stress that warps the flexible substrate; consequently, high-temperature sintering cannot be used on flexible substrates. Studies on the low-temperature sintering of CuNPs are limited [33,35]. Although CuNPs are protected by a blocking agent layer, over time, and depending on the type of blocking agent, they will still oxidize slowly, resulting in a very thin patina on the outside of the nanoparticles. Because CuNPs oxidize easily, especially under high temperature, sintering must be performed in an inert environment; studies on low-temperature sintering and the application of CuNPs to flexible substrates are also relatively limited. Therefore, in the current study, chemical reduction methods involving more environmentally friendly chemical agents (ascorbic acid) were used to prepare CuNPs and explore the effects of reaction temperature and reaction time on their preparation. The materials were analyzed using transmission electron microscopy (TEM), X-ray diffraction (XRD), and ultraviolet–visible spectrophotometry (UV–vis). The CuNPs were then formed into conductive ink and applied to a flexible polyethylene terephthalate (PET) substrate to investigate the effects of sintering time and sintering agent concentration on resistivity. The novelty and innovation of this study is the preparation of CuNPs with low toxicity and effective resistance to oxidation for the production of high-performance conductive ink that can be used on flexible substrates in low-temperature sintering environments.

## 2. Materials and Methods

### 2.1. Materials

Absolute ethanol (C_2_H_5_OH, purity ≥ 99.8%), copper (II) sulfate pentahydrate (CuSO_4_·5H_2_O, purity ≥ 98.0%), ascorbic acid (C_6_H_8_O_6_, purity ≥ 99.7%), and ammonia hydroxide (NH_3_·H_2_O, 35%) were purchased from Fisher Chemical (Pittsburgh, PA, USA) and used without further purification. Polyvinylpyrrolidone (PVP, purity ≥ 99.0%), ethylene glycol (C_2_H_4_(OH)_2_, purity ≥ 99.5%), formic acid (CH₂O₂, purity ≥ 95.0%), and sodium perchlorate (NaClO_4_, purity ≥ 99.0%) were obtained from Acros Organics (Geel, Belgium). Commercial wetting agent (type 2230) was used in this study, supplied by Marvel Chemical Co. (Taipei, Taiwan). PET ((C_10_H_8_O_4_)_n_) was purchased from Hsin Yun Co. (Taipei, Taiwan). All chemicals were analytical grade and used as received.

### 2.2. Preparing the CuNPs

**Figure 1:**
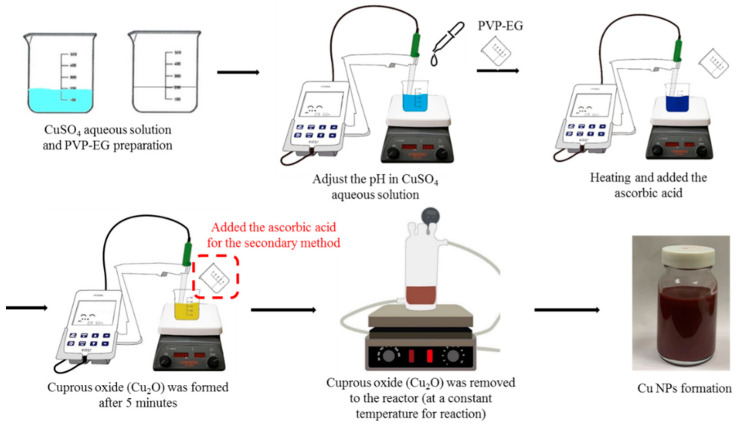
The preparation schematic diagram of CuNP.

This study involved the reduction and synthesis of CuNPs using ascorbic acid and one-factor testing with various variables to investigate the effects of each factor on their growth. The reduction methods were divided into primary and secondary [41]. The concentration of the reducing agent was expressed as the molar ratio of reducing agent to precursor copper sulfate. The control factors of the experiment were the reduction method, the reaction temperature, and the reduction time. Primary reduction involved preparing 30 mL of 0.02 M copper sulfate (CuSO_4_) aqueous solution and 30 mL of ethylene glycol containing 0.5 g of polyvinylpyrrolidone (PVP-EG) in cleaned and dried containers; the CuSO_4_ aqueous solution was placed in an induction mixer (350 rpm), and 35% ammonia (3–10 mL) was used to adjust the pH. The PVP-EG was then poured into the CuSO_4_ solution, and after the temperature was increased to 55 °C, various molar ratios (ascorbic acid/copper sulfate ((AA)/(CuSO_4_)) = 5, 10, 15, and 20) of ascorbic acid were added to the mixture. Finally, the mixture was maintained at a constant temperature (55 and 80 °C) for reaction. For secondary reduction, after the primary reduction was completed, a solution of ascorbic acid was added, and the solution was then maintained at a constant temperature (65, 70, 75, 80, 85, and 90 °C) for reaction. The preparation schematic diagram of CuNP is shown in Figure 1.

### 2.3. Flexible Substrate Pretreatment and Preparation of Cu Conductive Ink

First, the PET substrate was cut into 2 × 2 cm^2^ pieces that were placed in a beaker, which was then filled with a mixture of deionized water, ammonia, and sodium perchlorate. After being subjected to ultrasonic vibrations for 20 min, the chemically processed PET substrate was washed with ethanol and then dried. The CuNP solution underwent centrifugation at high speed, which formed sediments which were then washed several times with deionized water and collected through a glass funnel filter. The centrifugation speed was 13,000 rpm, for 10 min each time. After the CuNPs were formed, the CuNPs were combined with a solvent, a polymer matrix (2%), and a humectant (1%) before being subjected to ultrasonic vibration for 30 min to form copper ink, which was then used to coat the chemically processed PET substrates. After the substrate was coated with the copper ink, the ink was dried at a low temperature to ensure its adherence to the PET surface. It was then sintered at 130 °C for different durations (30, 60, and 90 min). Various concentrations (30, 50, 70, and 90% vol) of formic acid were then sprayed on the surface as a sintering aid, and thickness and electrical resistance were measured using an optical film thickness meter and a four-point probe. All the experiments were carried out in triplicate to confirm data reproducibility of the experimental results.

### 2.4. Characteristics of Nanoparticles and Electrical Properties of Conductive Ink Material

In this study, the CuNP solution samples without any rinsing were directly analyzed by UV–vis (Gensys 10 Series, Thermo Scientific, Waltham, MA, USA). The morphological properties of the CuNPs were studied using an H-7100 TEM (Hitachi, Tokyo, Japan). Measurement of the Cu conductive ink viscosity was carried out using a DV3T viscometer (Brookfield Engineering Laboratories, Middleboro, MA, USA). XRD (Empyrean, Malvern Panalytical, Malvern, UK) was used to further characterize the CuNPs. The surface tension of Cu conductive ink was assessed using a Model 100SB device (Sindatek Instruments, New Taipei, Taiwan). Fourier-transform infrared (FTIR) spectroscopy (FT/IR-6000, Jasco International Co., Tokyo, Japan) was used to confirm the functional groups change of PET before and after surface modification, and atomic force microscopy (AFM; XE-100, Park Scientific Instruments, Sunnyvale, CA, USA) was used to measure the surface roughness. Finally, the electrical resistivity of the patterns was measured using a Surfcorder ET3000 microfigure measuring instrument (Kosaka Laboratory, Tokyo, Japan) and a Keithley 2000-EM4P four-point probe analyzer (Tektronix, Beaverton, OR, USA).

## 3. Results and Discussion

### 3.1. Synthesis of Cu Nanoparticles

#### 3.1.1. Comparison of Reduction Methods

**Figure 2:**
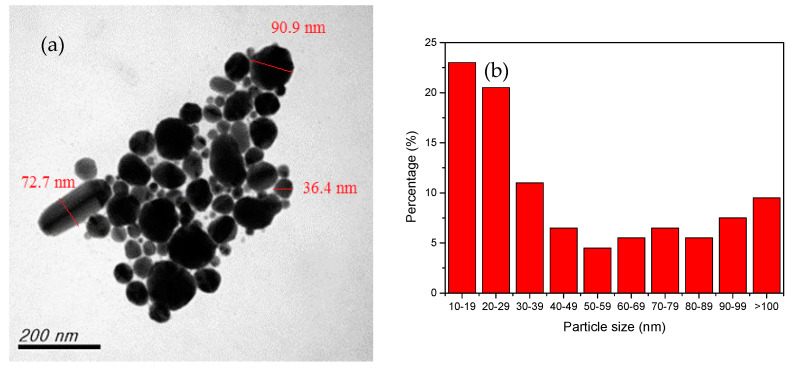
Primary reduction when (AA)/(CuSO_4_) = 15 and pH = 10.5. (**a**) TEM. (**b**) Particle size distribution.

**Figure 3:**
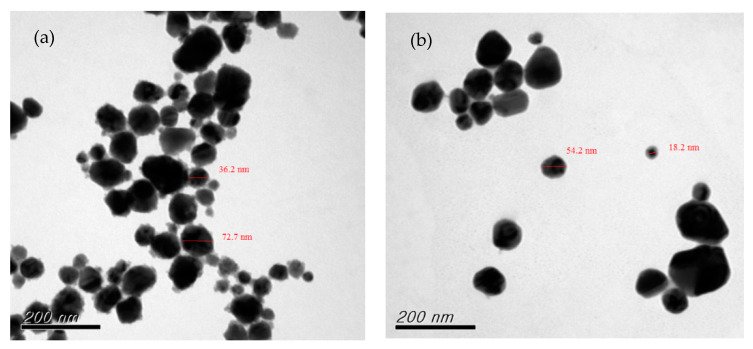
Primary reduction when (AA)/(CuSO_4_) = 15: (**a**) pH = 11; (**b**) pH = 11.5.

**Figure 4:**
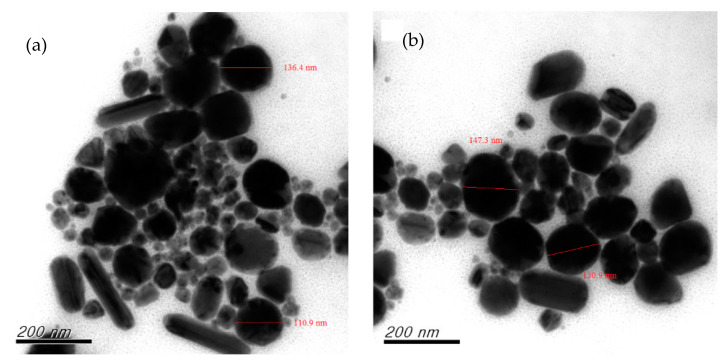
Primary reduction when (AA)/(CuSO_4_) = 20: (**a**) pH = 10.5; (**b**) pH = 11.

A reducing agent is a substance that loses electrons during the oxidation reduction reaction, providing electrons to the reduced substance [29,35]. The reducing agent is one of the most important factors influencing the growth and aggregation of CuNPs. It can be controlled not only by which agent is selected and its concentration, but also by the method of dosage addition or secondary reduction of the chemical reduction process [5,42,43]. In the current study, the effects of the reducing agent concentration were explored by adjusting the molar ratio of the agent to that of the precursor copper sulfate ((AA)/(CuSO_4_)). The results of preparing CuNPs through primary reduction when (AA)/(CuSO_4_) = 15 and pH = 10.5 are presented in Figure 2a, showing that the particles differed greatly in size distribution and exhibited rod-like and uneven shapes. The particle size distribution is presented in Figure 2b. When (AA)/(CuSO_4_) = 15 and pH = 11 (Figure 3a), only a few particles were quadrilateral, whereas several were large and exhibited serious aggregation and inconsistent shapes. When (AA)/(CuSO_4_) = 15 and pH = 11.5 (Figure 3b), most particles were round, oval, or nearly round. In our past research [42], the result indicated that appropriately increasing the concentration of the reducing agent can result in smaller, more uniform particles. In the current study, primary reduction ((AA)/(CuSO_4_) = 20 and pH = 10.5; Figure 4a) resulted in numerous large, rod-shaped nanoparticles. Figure 4b presents the results of CuNPs when pH = 11 and the reducing agent ratio was the same. Despite the more favorable crystallization effect, the proportion of larger particles was higher.

**Figure 5:**
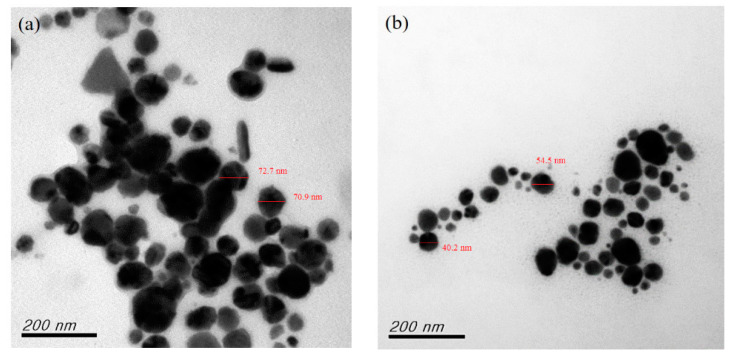
TEM of secondary reduction: (**a**) (AA)/(CuSO_4_) = 10; (**b**) (AA)/(CuSO_4_) = 15.

The results demonstrate that the pH and reducing agent concentration are the key factors affecting the growth of CuNPs; however, the effects of primary reduction were found to be unsatisfactory. Specifically, when (AA)/(CuSO_4_) = 15 and pH = 10.5, the particle sizes were not uniform, and increasing the pH or reducing agent concentration resulted in flaws such as particle aggregation and larger particles. Secondary reduction was also used to prepare CuNPs, with the second reaction occurring under the same molar ratio as that of (AA)/(CuSO_4_). When the molar ratio was 10, the resulting CuNPs were larger and exhibited a degree of fusing. When the (AA)/(CuSO_4_) molar ratio was 15, the CuNPs were smaller, round, and more dispersed (Figure 5). Therefore, this molar ratio was fixed in subsequent experiments. Preparation by secondary reduction resulted in smaller and more evenly dispersed CuNPs, possibly because the excessive amount of ascorbic acid present during secondary reduction acts as a stabilizer. Furthermore, the dehydroascorbic acid that formed after ascorbic acid oxidation contained hydrogen bonds, which can prevent the aggregation of nanoparticles [44].

#### 3.1.2. Effect of Reaction Temperature

**Figure 6:**
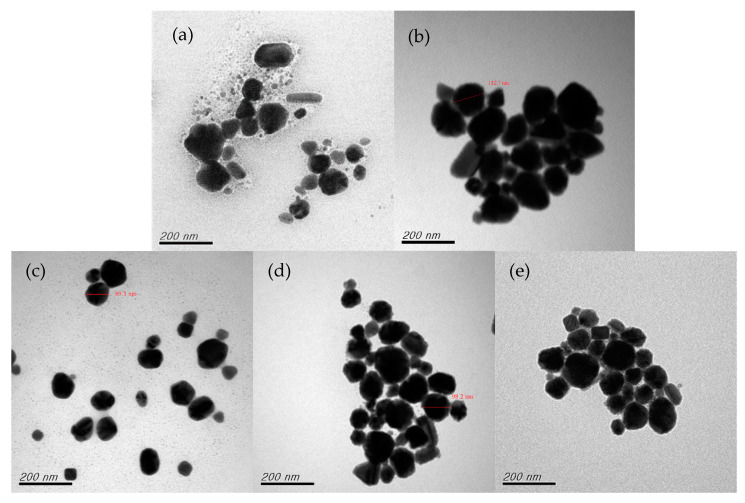
TEM images of CuNPs under secondary reduction at temperatures of (**a**) 65 °C, (**b**) 75 °C, (**c**) 80 °C, (**d**) 85 °C, and (**e**) 90 °C.

The reaction temperature is a key factor in the chemical reduction process [45,46]. With the molar ratio fixed at 15, the effect of reaction temperature (65, 70, 75, 80, 85, and 90 °C) on the growth of the CuNPs was examined. Figure 6 presents the results of preparing CuNPs by using a secondary reduction method under various temperatures. When the reaction temperature was 65 °C, several nanonuclei were not fully formed, indicating an insufficient reaction temperature. When the reaction temperature was 75 °C, no copper nanonuclei remained; however, some mild aggregation was observed. When the reaction temperature was 80 °C, the particles were small and well dispersed. When the reaction temperature exceeded 80 °C, the resulting CuNPs were similar in size, but they exhibited some aggregation. These results are consistent with those of relevant research, because under high temperatures, ethylene glycol displays reducing effects (polyol reduction); the OH base in ethylene glycol may react with copper to form a protective layer, reducing the viscosity and limiting the growth and aggregation of CuNPs [46]. However, when the temperature is increased, the reaction system interferes with the stabilizers, which leads to failure to cover the surface of the nanoparticles, resulting in CuNP agglomeration.

**Figure 7:**
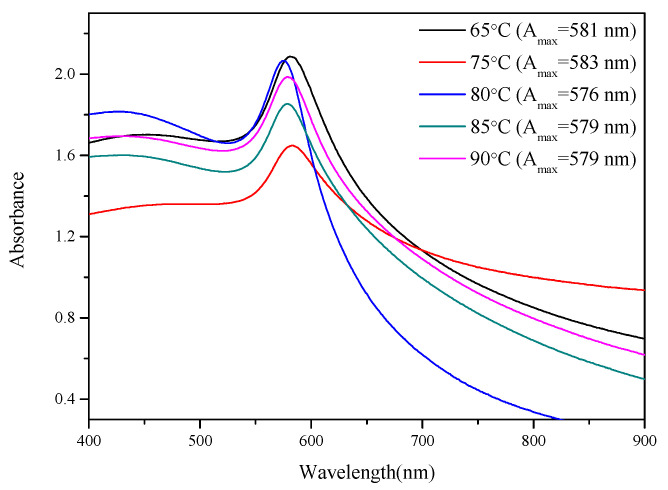
UV–vis spectra of CuNPs synthesized under secondary reduction at temperatures of 65 °C, 75 °C, 80 °C, 85 °C, and 90 °C.

Furthermore, when the wavelength position of the strongest peak (A_max_) of the nanoparticles on the UV–vis spectrum becomes shorter, the CuNPs are smaller (blue shift); when the wavelength position of the strongest peak becomes longer, the CuNPs are larger (red shift) [47]. The peak value can also be used to determine the relative amount of CuNPs, according to the Beer–Lambert law [48]. The UV–vis spectrum (Figure 7) also demonstrates that for CuNPs formed at 80 °C, the wavelength position of the strongest peak was the shortest; therefore, 80 °C was selected as the reaction temperature for subsequent reactions.

#### 3.1.3. Effects of Reaction Time

**Figure 8:**
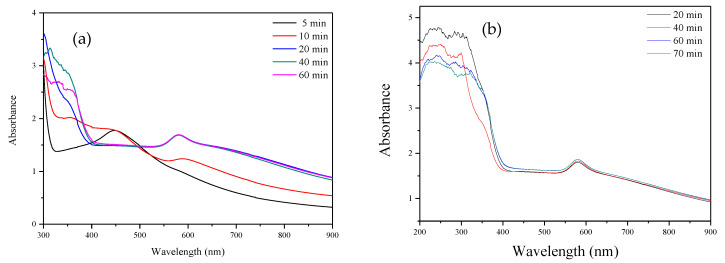
(**a**) UV–vis spectrogram of CuNPs synthesized under different reaction times. (**b**) UV–vis spectrogram of ascorbic acid when synthesizing CuNPs under different reaction times.

Reaction time also affects metallic nanoparticle generation in the chemical reduction methods [49,50]. To understand the relationship between synthesized CuNPs and reaction time, UV–vis spectrograms were used to visualize the changes in peak intensity and the wavelength position of the tallest peak over time. Figure 8a displays the UV–vis spectrogram for reaction times from 5 min to 1 h. When the reaction time was 5 min, the highest peak was positioned at a wavelength of 447 nm, indicating that cuprous oxide (Cu_2_O) had formed. When the reaction time was 10 min, the peak for copper (550 to 610 nm) gradually formed, whereas the peak for Cu_2_O gradually became less evident, indicating that copper was forming [51]. From 20 to 40 min, the highest peaks were all positioned at 581 nm, and at 50 min, the wavelength position of the highest peak blue-shifted to 580 nm and remained there until 60 min. This indicates that the particles continued to become smaller. Furthermore, the wavelength position of the highest peak for ascorbic acid was at approximately 266 nm, and over time, the preceding peak decreased in intensity, indicating that the ascorbic acid continued to deplete over time. To confirm when the ascorbic acid was no longer being depleted, the range of the UV–vis spectrogram was set to 200–900 nm (Figure 8b). In the 60–70 min interval, the wavelength position of the copper peak was approximately 580 nm and the peak value of the preceding 266 nm position reached equilibrium. This indicates that the preparation reaction time in the current study was approximately 1 h.

**Figure 9:**
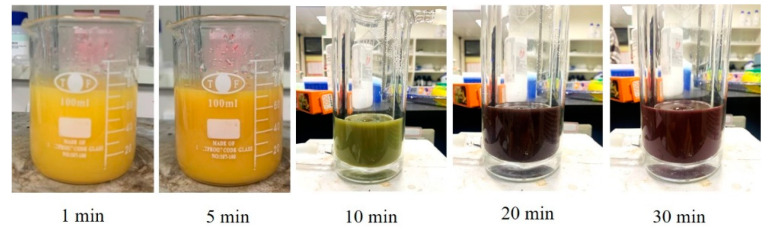
Color changes according to reaction times when synthesizing CuNPs.

**Figure 10:**
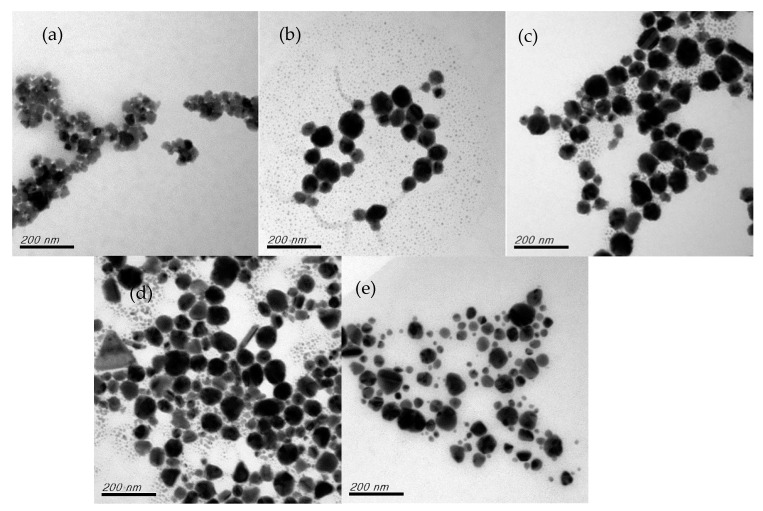
Changes in shape under reaction times of (**a**) 5 min, (**b**) 10 min, (**c**) 30 min, (**d**) 40 min, and (**e**) 60 min.

Figure 9 depicts the color changes in the CuNPs formed under different reaction times. Over time, the CuNPs appeared yellow, green, brown, and, finally, brown-red. A yellow color appeared immediately after the addition of ascorbic acid. The small increase in absorbance of 580 nm (Figure 8b) turned the yellow color to brown-red, indicating the formation of a nanoclustor of zero valent Cu under the reduction reaction [46]. After 30 min, the color remained brown-red, but based on the results of the UV–vis spectrum analysis, the reaction time must be 60 min to be considered complete. Figure 10 displays the changes in shape over time during CuNP reduction. At 5 min, the particles were aggregated, but cuprous oxide was unset; at 10 min, CuNPs began to form, but they were surrounded by numerous copper nanonuclei. From then until 40 min, some copper nanonuclei remained and some small rod-shaped particles and some triangular particles were present until they disappeared at 60 min. CuNPs of other shapes were speculated to be the predecessors of round CuNPs, which gained additional sides and became round as the reaction time increased.

**Figure 11:**
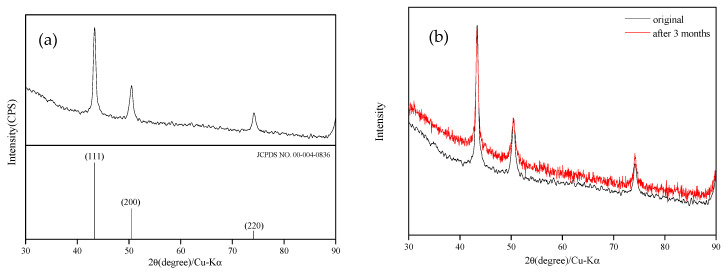
XRD spectrograms of (**a**) CuNPs and (**b**) CuNP solution after 3 months.

XRD analysis was performed on the prepared nanoparticles (Figure 11a). The results of the analysis revealed three characteristic peaks: 43.3°, 50.4°, and 74.08°. These results are consistent with those of XRD JCPDS (Joint Committee on Powder Diffraction Standards) No. 00-004-0836, confirming that they were copper crystal structures [10,14]. To determine the stability of the CuNP solution, the samples were stored under an ambient atmosphere for 3 months and then analyzed again for comparison (Figure 11b). The peak values of the three characteristic peaks remained strong, confirming that samples such as those prepared in the current study can be stored for at least 3 months.

### 3.2. Cu Conductive Sheets

#### 3.2.1. Cu Ink Preparation and Surface Modification of PET Substrate

Conductive ink, as one of the key raw materials in flexible electronics, can determine the rate, direction, and cost of flexible electronics development. Conductive ink is a multi-component system (e.g., containing additive, conductive filler, and solvent) [2]. PVP, a water-soluble polymer that is less restricted in its use than polymers that are only soluble in organic humectants, was used in the polymer matrix. A small amount of PVP added to water can increase the viscosity of the solution. Furthermore, the high molecular weight of the polymer also helps the copper ink adhere to the flexible substrate [52]. PVP, because of its unique lactam structure, has both hydrophilic and lipophilic properties and can thus form a film with strong binding force on plastic [52,53]. Furthermore, PVP is also often used as a surface dispersant that prevents ink from precipitating [35,54]. Because plasma contains both solids and liquids, commercial nanojet humectants were added to enable the heterogeneous liquid to emulsify and evenly disperse; the copper ink appeared even after the plasma was left alone for several days. The next day, stratification was observed in the copper ink without humectant.

**Figure 12:**
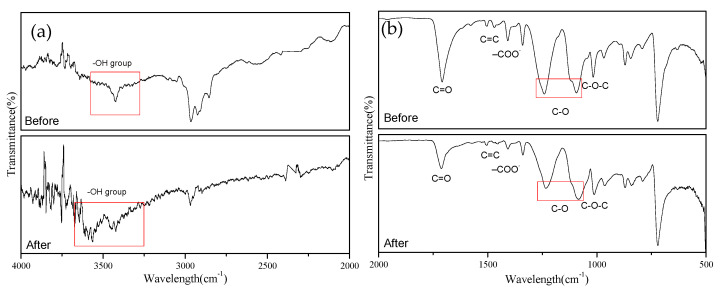
FTIR images of PET before and after surface modification: (**a**) 2000–4000 cm^−1^; (**b**) 500–2000 cm^−1^.

PET plastic substrate is smooth. To increase interfacial bonding, the surface must first be modified [55,56]. In the current study, the surface was modified chemically: the PET substrate was immersed in an oxidant (sodium perchlorate) and an aqueous solution of ammonia to oxidize and break the molecular chains on the surface of the PET film, thereby increasing its surface roughness and introducing polar groups. Figure 12 displays FTIR images of the PET before and after surface modification. Significant changes in the absorbance peak were observed after treatment. Based on the stretching vibrations of the OH group appearing in the 3700–3100^−1^ range, the absorption peak of the OH polar group intensified after treatment. This may be because the alkaline aqueous solution of sodium perchlorate contains sodium hypochlorite, hypochlorous acid, and NaOH. Sodium hypochlorite can be hydrolyzed into NaOH, and when the PET was treated with NaOH, the number of OH groups increased [57]. Figure 12b illustrates that after PET treatment, some of the main absorption peaks weakened, possibly because the PET polymers were degraded by the alkaline treatment and HOCl oxidation [58].

**Figure 13:**
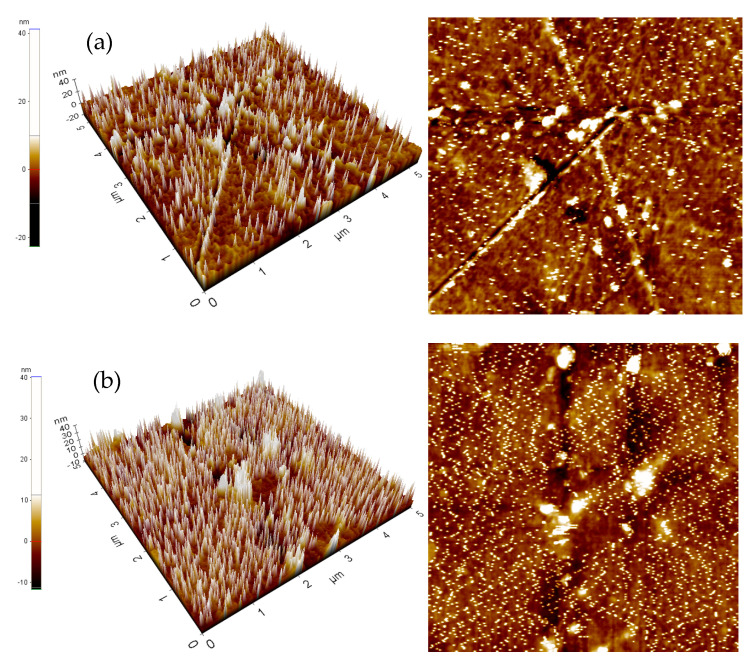
Three-dimensional AFM high and phase images of PET substrate (**a**) before and (**b**) after surface modification.

**Figure 14:**
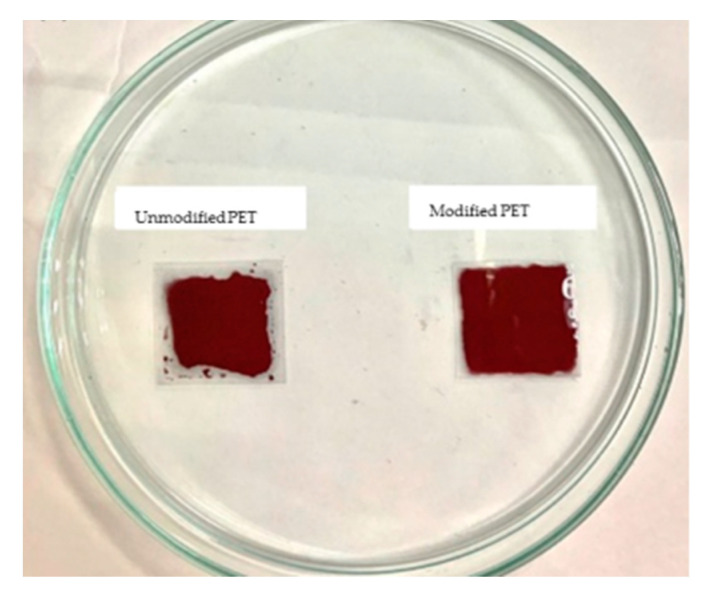
Adhesive status on modified and unmodified PET substrates.

Furthermore, surface undulations of the samples were detected using AFM, and the results are presented in Figure 13. The roughness of the modified surface (Ra = 3.728 nm) was greater than that of the unmodified sample (Ra = 3.018 nm), confirming that the surface area of the PET film was effectively increased, thereby improving the binding force between it and the copper ink [59]. The adhesive capacity was tested by dripping 0.1 mL of copper ink on modified and unmodified PET substrates and spreading it evenly outward. The results revealed that the copper ink could not evenly adhere to the unmodified PET substrate and shrank toward the center (Figure 14). This became more evident over time. On the modified PET substrate, the copper ink demonstrated satisfactory ductility and the ability to adhere to the target area.

#### 3.2.2. Electric Resistivity of Conductive Sheets

**Figure 15:**
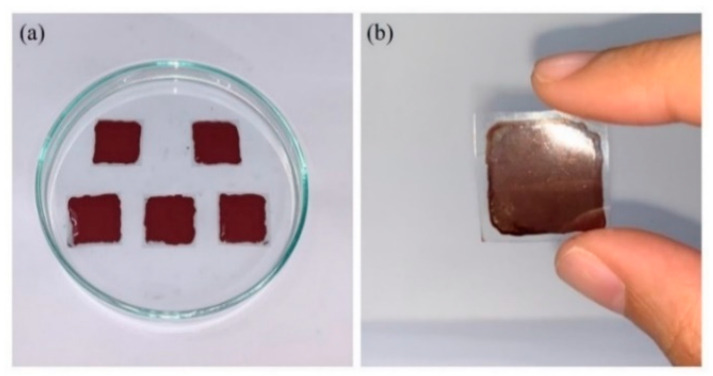
(**a**) PET substrates painted with conductive plasma. (**b**) Sample after sintering.

Squares of copper ink of the same volume were drawn on each substrate with a pipette, as depicted in Figure 15a. Next, the samples were placed in a vacuum oven at 80 °C for 30 min. Formic acid was then sprayed on them, and they were placed in an oven for sintering at 130 °C. The resulting samples exhibited a visible metallic sheen, as shown in Figure 15b.

**Figure 16:**
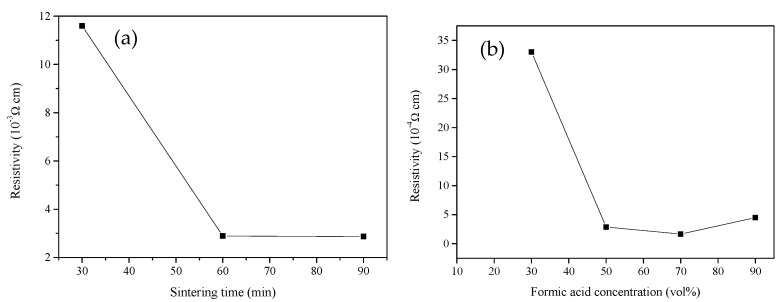
(**a**) Resistivity with sintering time. (**b**) Effects of different formic acid concentrations on resistivity.

Because the oven is not a complete vacuum, an oxidized microlayer formed on the copper surface. Further, because formic acid has decomposition properties that can transform cupric oxide into copper (II) formate, this salt compound decomposes easily after being heated, and after decomposition forms into copper [60]. The copper-conductive sheets sintered at a low temperature of 130 °C were treated with formic acid. A thin film analyzer was used to measure the average thickness of the film formed on the surface of PET, which was 3.96 μm. Currently, ideal resistivity is approximately 10^–4^–10^–3^ Ω·cm; the resistivity resulting from various sintering times (30, 60, and 90 min) with 50% formic acid and a sintering temperature of 130 °C is presented in Figure 16a. The results indicate that after 30, 60, and 90 min of sintering, resistivity was 1.6 × 10^−2^, 2.89 × 10^−3^, and 2.87 × 10^−3^ Ω·cm, respectively. Figure 16b presents the results of resistivity after 60 min of sintering at 130 °C and under different concentrations of formic acid. When the concentration of formic acid was 10%, no resistivity was detected. When the concentration was 30 and 70%, resistivity was 3.3 × 10^−2^ and 1.67 × 10^−3^ Ω·cm, respectively. When the concentration of formic acid was 90%, resistivity began to increase.

## 4. Conclusions

The current study explored the synthesis of nanoparticles by using primary chemical reduction method and secondary chemical reduction method. The results revealed that the CuNPs that formed through secondary chemical reduction method were better than primary chemical reduction method, as the CuNPs formed through secondary chemical reduction method were smaller and uniform. When the reaction temperature of the secondary reduction was 80 °C, the resulting CuNPs were fully reduced and more dispersed. The conditions for synthesizing CuNPs in the current study were a reaction temperature of 80 °C, a precursor pH of 11, an (AA)/(CuSO_4_) molar ratio of 15:1, and a reaction time of 60 min. Under these conditions, CuNPs with an average particle diameter of 43 nm can be synthesized.

The CuNPs prepared in this study and the conductive copper plastic prepared with PVP, water, and humectants were coated onto PET substrates with a pipette and subsequently sintered at 130 °C for 60 min with a 70% concentration of formic acid to obtain the lowest resistivity (1.67 × 10^−3^ Ω·cm). Conductive metal nanoparticles are vital in electronic and optoelectronic component applications. In addition to being highly conductive, their mechanical properties render them suitable for flexible electronics. Conductive metal nanoparticles are vital in electronic and optoelectronic component applications. In addition to being highly conductive, their mechanical properties make them suitable for flexible electronics. The CuNPs prepared in the current study are stable and resistant to oxidation and therefore have potential for practical applications.

## Data Availability

Not applicable.

## References

[1] Arrese J., Vescio G., Xuriguera E., Medina-Rodriguez B., Cornet A., Cirera A. (2017). Flexible hybrid circuit fully inkjet-printed: Surface mount devices assembled by silver nanoparticles-based inkjet ink. J. Appl. Phys..

[2] Shao W., Li G., Zhu P., Zhang Y., Ouyang Q., Sun R., Chen C., Wong C.P. (2018). Facile synthesis of low temperature sintering Ag nanopaticles for printed flexible electronics. J. Mater. Sci Mater. Electron..

[3] Dai X., Xu W., Zhang T., Shi H., Wang T. (2019). Room temperature sintering of Cu-Ag core-shell nanoparticles conductive inks for printed electronics. Chem. Eng. J..

[4] Cao L., Bai X., Lin Z., Zhang P., Deng S., Du X., Li W. (2017). The preparation of Ag nanoparticle and ink used for inkjet printing of paper based conductive patterns. Materials.

[5] Jamkhande P.G., Ghule N.E., Bamer A.H., Kalaskar M.G. (2019). Metal nanoparticles synthesis: An overview on methods of preparation, advantages and disadvantages, and applications. J. Drug Deliv. Sci. Technol..

[6] Begletsova N., Selifonova E., Chumakov A., Al-Alwani A., Zakharevich A., Chernova R., Glukhovskoy E. (2018). Chemical synthesis of copper nanoparticles in aqueous solutions in the presence of anionic surfactant sodium dodecyl sulfate. Colloids Surf. A Phys. Eng. Asp..

[7] Gawande M.B., Goswami A., Felpin F.-X., Asefa T., Huang X., Silva R., Zou X., Zboril R., Varma R.S. (2016). Cu and Cu-based nanoparticles: Synthesis and applications in catalysis. Chem. Rev..

[8] Seo Y., Hwang J., Lee E., Kim Y.J., Lee K., Park C., Choi Y., Jeon H., Choi J. (2018). Engineering copper nanoparticles synthesized on the surface of carbon nanotubes for anti-microbial and anti-biofilm applications. Nanoscale.

[9] Tomotoshi D., Kawasaki H. (2020). Surface and Interface Designs in Copper-Based Conductive Inks for Printed/Flexible Electronics. Nanomaterials.

[10] Li W., Cen Q., Li W., Zhao Z., Yang W., Li Y., Chen M., Yang G., Yang J. (2020). A green method for synthesizing novel nanoparticles and their application in flexible conductive patterns. J. Mater..

[11] Uzair B., Liaqat A., Iqbal H., Menaa B., Razzaq A., Thiripuranathar G., Fatima Rana N., Menaa F. (2020). Green and cost-effective synthesis of metallic nanoparticles by algae: Safe methods for translational medicine. Bioengineering.

[12] Thakur P.K., Verma V. (2021). A Review on green synthesis, characterization and anticancer application of metallic nanoparticles. Appl. Biochem. Biotechnol..

[13] Patra D., Kurdi R.E. (2021). Curcumin as a novel reducing and stabilizing agent for the green synthesis of metallic nanoparticles. Green Chem. Lett. Rev..

[14] Jardon-Maximino N., Perez-Alvarez M., Cadenas-Pliego G., Lugo-Uribe L.E., Cabello-Alvarado C., Mata-Padilla J.M., Barriga-Castro E.D. (2021). Synthesis of copper nanoparticles stabilized with organic ligands and their antimicrobial properties. Polymers.

[15] Brycki B., Szulc A., Babkova M. (2021). Synthesis of silver nanoparticles with gemini surfactants as efficient capping and stabilizing agents. Appl. Sci..

[16] Harada M., Yamamoto M., Sakata M. (2020). Temperature dependence on the size control of palladium nanoparticles by chemical reduction in nonionic surfactant/ionic liquid hybrid systems. J. Mol. Liq..

[17] Zahoor M., Nazir N., Iftikhar M., Naz S., Zekker I., Burlakovs J., Uddin F., Kamran A.W., Kallistova A., Pimenov N. (2021). A review on silver nanoparticles: Classification, various methods of synthesis, and their potential roles in biomedical applications and water treatment. Water.

[18] Ielo I., Rando G., Giacobello F., Sfameni S., Castellano A., Galletta M., Drommi D., Rosace G., Plutino M.R. (2021). Synthesis, chemical–physical characterization, and biomedical applications of functional gold nanoparticles: A review. Molecules.

[19] Thanh N.T., Maclean N., Mahiddine S. (2014). Mechanisms of nucleation and growth of nanoparticles in solution. Chem. Rev..

[20] Firdhouse M.J., Lalitha P. (2015). Biosynthesis of silver nanoparticles and its applications. J. Nanotechnol..

[21] Wang Z., Liang X., Zhao T., Hu Y., Zhu P., Sun R. (2017). Facile synthesis of monodisperse silver nanoparticles for screen printing conductive inks. J. Mater. Sci. Mater. Electron..

[22] Fernandes I.J., Aroche A.F., Schuck A., Lamberty F., Peter C.R., Hasenkamp W., Rocha T.L.A.C. (2020). Silver nanoparticle conductive inks: Synthesis, characterization, and fabrication of inkjet-printed flexible electrodes. Sci. Rep..

[23] Mirzaei A., Janghorban K., Hashemi B., Bonyani M., Leonardi S.G., Neri G. (2017). Characterization and optical studies of PVP-capped silver nanoparticles. J. Nanostructure Chem..

[24] Soltys L., Olkhovyy O., Tatarchuk T., Naushad M. (2021). Green synthesis of metal and metal oxide nanoparticles: Principles of green chemistry and raw materials. Magnetochemistry.

[25] Dai X., Xu W., Zhang T., Wang T. (2018). Self-reducible Cu nanoparticles for conductive inks. Ind. Eng. Chem. Res..

[26] Pozdnyakov A.S., Emellyanov A.I., Korzhova S.A., Kuznetsova N.P., Bolgova Y.I., Trofimova O.M., Semenova T.A., Prozorova G.F. (2021). Green synthesis of stable nanocomposites containing copper nanoparticles incorporated in poly-N-vinylimidazole. Polymers.

[27] Ghadiri A.M., Rabiee N., Bagherzadeh M., Kiani M., Fatahi Y., Di-Bartolomeo A., Dinarvand R., Webster T.J. (2020). Green synthesis of CuO- and Cu_2_O-NPs in assistance with high-gravity: The flowering of nanobiotechnology. Nanotechnology.

[28] Benassai E., Bubba M.D., Ancillotti C., Colzi I., Gonnelli C., Calisi N., Salvatici M.C., Casalone E., Ristori S. (2021). Green and cost-effective synthesis of copper nanoparticles by extracts of non-edible and waste plant materials from vaccinium species: Characterization and antimicrobial activity. Mater. Sci. Eng. C.

[29] Nagar N., Devra V. (2018). Green synthesis and characterization of copper nanoparticles using azadirachta indica leaves. Mater. Chem. Phys..

[30] Duan H.H., Wang D.S., Li Y.D. (2015). Green chemistry for nanoparticle synthesis. Chem. Soc. Rev..

[31] Jayarambabu N., Akshaykranth A., Rao T.V., Rao K.V., Kumar R.R. (2020). Green synthesis of Cu nanoparticles using curcuma longa extract and their application in antimicrobial activity. Mater. Lett..

[32] Zhang Y., Zhu P., Li G., Zhao T., Fu X., Sun R., Zhou F., Wong C. (2014). Facile preparation of monodisperse, impurity-free, and antioxidation copper nanoparticles on a large scale for application in conductive ink. ACS Appl. Mater. Interfaces.

[33] Abhinav K.V., Rao R.V.K., Karthik P.S., Singh S.P. (2015). Copper conductive inks: Synthesis and utilization in flexible electronics. RSC Adv..

[34] Sarwar N., Humayoun U.B., Kumar M., Zaidi S.F.A., Yoo J.H., Ali N., Jeong D.I., Lee J.H., Yoon D.H. (2021). Citric acid mediated green synthesis of copper nanoparticles using cinnamon bark extract and its multifaceted applications. J. Clean. Prod..

[35] Yang W., Liu C., Zhang Z., Liu Y., Nie S. (2013). Preparation and conductive mechanism of copper nanoparticles ink. J. Mater. Sci. Mater. Electron..

[36] Li W., Sun Q., Li L., Jiu J., Liu X.Y., Kanehara M., Minari T., Suganuma K. (2020). The rise of conductive copper inks: Challenges and perspectives. Appl. Mater. Today.

[37] Magdassi S., Grouchko M., Kamyshny A. (2010). Copper nanoparticles for printed electronics: Routes towards achieving oxidation stability. Materials.

[38] Reverberi A.P., Salerno M., Lauciello S., Fabiano B. (2016). Synthesis of copper nanoparticles in ethylene glycol by chemical reduction with vanadium (+2) salts. Materials.

[39] El-Berry M.F., Sadeek S.A., Abdalla A.M., Nassar M.Y. (2021). Facile, controllable, chemical reduction synthesis of copper nanostructures utilizing different capping agents. Inorg. Nano-Met. Chem..

[40] Wu C.J., Chen S.M., Sheng Y.J., Tsao H.K. (2014). Anti-oxidative copper nanoparticles and their conductive assembly sintered at room temperature. J. Taiwan Inst. Chem. Eng..

[41] Yang J.G., Okamoto T., Ichino R., Satake S., Okido M. (2006). A Simple Way for Preparing Antioxidation Nano-copper Powders. Chem. Lett..

[42] Hong G.B., Luo Y.H., Chuang K.J., Ma C.M. (2021). Preparing and applying silver nanoparticles in conductive ink and inkjet painting. J. Nanosci. Nanotechnol..

[43] Yaqoob A.A., Umar K., Ibrahim M.N.M. (2020). Silver nanoparticles: Various methods of synthesis, size affecting factors and their potential applications–a review. Appl. Nanosci..

[44] Xiong J., Wang Y., Xue Q., Wu X. (2011). Synthesis of highly stable dispersions of nanosized copper particles using L-ascorbic acid. Green Chem..

[45] Rostami-Tapeh-Esmaeil E., Golshan M., Salami-Kalajahi M., Roghani-Mamaqani H. (2021). Synthesis of copper and copper oxide nanoparticles with different morphologies using aniline as reducing agent. Solid State Commun..

[46] Luo C., Zhang Y., Zeng X., Zeng Y., Wang Y. (2005). The role of poly(ethylene glycol) in the formation of silver nanoparticles. J. Colloid Interface Sci..

[47] Soomro R.A., Sherazi S.T.H., Sirajuddin, Sherazi S.T.H., Memon N., Shah M.R., Kalwar N.H., Hallam K.R., Shah A. (2014). Synthesis of air stable copper nanoparticles and their use in catalysis. Adv. Mat. Lett..

[48] Liu X., Atwater M., Wang J., Huo Q. (2007). Extinction coefficient of gold nanoparticles with different sizes and different capping ligands. Colloids Surf B: Biointerfaces.

[49] Restrepo C.V., Villa C.V. (2021). Synthesis of silver nanoparticles, influence of capping agents, and dependence on size and shape: A review. Environ. Nanotechnol. Monit. Manag..

[50] Ranoszek-Soliwoda K., Tomaszewska E., Socha E., Krzyczmonik P., Ignaczak A., Orlowski P., Krzyżowska M., Celichowski G., Grobelny J. (2017). The role of tannic acid and sodium citrate in the synthesis of silver nanoparticles. J. Nanoparticle Res..

[51] Gurav P., Naik S.S., Ansari K., Srinath S., Kishore K.A., Setty Y.P., Sonawane S. (2014). Stable colloidal copper nanoparticles for a nanofluid: Production and application. Colloids Surf. A Physicochem. Eng. Asp..

[52] Huaman J.L.C., Urushizaki I., Jeyadevan B. (2018). Large-scale Cu nanowire synthesis by PVP-ethylene glycol route. J. Nanomater..

[53] Yokoyama S., Nozaki J., Motomiya K., Tsukahara N., Takahashi H. (2020). Strong adhesion of polyvinylpyrrolidone-coated copper nanoparticles on various substrates fabricated from well-dispersed copper nanoparticle inks. Colloids Surf. A Physicochem. Eng. Asp..

[54] Jun H.Y., Lee E.L., Ryu S.O. (2020). Synthesis and characterization of copper ink and direct printing of copper patterns by inkjet printing for electronic devices. Curr. Appl. Phys..

[55] Zhang Y., Zhang T., Shi H.B., Liu Q., Wang T. (2021). Fabrication of flexible copper patterns by electroless plating with copper nanoparticles as seeds. Appl. Surf. Sci..

[56] Zheng Z., Ren L., Feng W., Zhai Z., Wang Y. (2012). Surface characterization of polyethylene terephthalate films treated by ammonia low-temperature plasma. Appl. Surf. Sci..

[57] Pereira A.P.D.S., Silva M.H.P.D., Junior É.P.L., Paula A.D.S., Tommasini F.J. (2017). Processing and characterization of PET composites reinforced with geopolymer concrete waste. Mater. Res..

[58] Kim E.Y., Kong J.S., An S.K., Kim H.D. (2000). Surface modification of polymers and improvement of the adhesion between evaporated copper metal film and a polymer. I. Chemical modification of PET. J. Adhes. Sci. Technol..

[59] Yang W.D., Wang C.H., Arrighi V., Liu C.Y., Watson D. (2015). Microstructure and electrical property of copper films on a flexible substrate formed by an organic ink with 9.6 % of Cu content. J. Mater. Sci. Mater. Electron..

[60] Woo K., Kim Y., Lee B., Kim J., Moon J. (2011). Effect of carboxylic acid on sintering of inkjet-printed copper nanoparticulate films. ACS Appl. Mater. Interfaces.

